# Brain plasticity and motor practice in cognitive aging

**DOI:** 10.3389/fnagi.2014.00031

**Published:** 2014-03-10

**Authors:** Liuyang Cai, John S. Y. Chan, Jin H. Yan, Kaiping Peng

**Affiliations:** ^1^Department of Psychology, Tsinghua UniversityBeijing, China; ^2^Department of Psychology, The Chinese University of Hong KongHong Kong, China; ^3^Institute of Affective and Social Neuroscience, Shenzhen UniversityShenzhen, China

**Keywords:** cognitive development, geriatric rehabilitation, motor performance, movement-dependent neural plasticity, skill acquisition

## Abstract

For more than two decades, there have been extensive studies of experience-based neural plasticity exploring effective applications of brain plasticity for cognitive and motor development. Research suggests that human brains continuously undergo structural reorganization and functional changes in response to stimulations or training. From a developmental point of view, the assumption of lifespan brain plasticity has been extended to older adults in terms of the benefits of cognitive training and physical therapy. To summarize recent developments, *first*, we introduce the concept of neural plasticity from a developmental perspective. *Secondly*, we note that motor learning often refers to deliberate practice and the resulting performance enhancement and adaptability. We discuss the close interplay between neural plasticity, motor learning and cognitive aging. *Thirdly*, we review research on motor skill acquisition in older adults with, and without, impairments relative to aging-related cognitive decline. *Finally*, to enhance future research and application, we highlight the implications of neural plasticity in skills learning and cognitive rehabilitation for the aging population.

## Introduction

Functional decline is evident in cognitive, motor, social, psychological, physical domains. Older adults often experience greater anxiety, poorer memory and attention, slower processing speeds; motor control and learning capabilities decreased; greater behavioral variability is usually observed. These areas of decline collectively cause neurodegenerative disorders like mild cognitive impairment (MCI), Alzheimer’s disease (AD), or AD-related dementia. The compromised functionality reduces the quality of life and causes great concerns in the geriatric and gerontological communities. However, functional deterioration is not inevitable. Behavioral improvements are likely to occur while having an active lifestyle that is a key to reduce the negative impact of cognitive-motor aging on daily functions and to enhance the quality of life. The development of effective diagnostic tools, better intervention strategies and technologies is of the utmost importance for geriatric researchers and practitioners (Yan and Zhou, 2009; Ren et al., 2013).

The gradual age-related decline in neural-behavioral functionality considerably impacts adults aged 65 or older (Yan et al., 1998, 2000). It is encouraging, however, that motor, physical and mental activities often promote *brain fitness* or* health* (*e.g*., the capacity to learn, improve and meet various cognitive demands) and *motor abilities* for coping with daily challenges. Actively engaging in physical, motor or intellectual exercises, or deliberately receiving multisensory stimulations, can prevent functional decline and preserve cognitive functions. In general, cognitive and motor performance deteriorates considerably as a result of an inactive lifestyle, biological aging and cognitive impairments (Yan, 1999; Teri et al., 2003; Yan and Dick, 2006; Liu et al., 2013).

Although the exact role of regular physical and mental exercise for *brain fitness* is unclear, there is a consensus that cognitive-motor activities or stimulations facilitate neuro-protection (Hillman et al., 2008; Taubert et al., 2010)*.* One of the processes is to strengthen *experience-based neural plasticity* through deliberate practice (Rakic, 2002; Trachtenberg et al., 2002; Colcombe et al., 2004; DeFelipe, 2006). Structural and functional changes (neural reorganizations) of the brain include the development of new neurons (neurogenesis), the generation of new glial cells (gliogenesis), strengthening of existing connections or growth of new synapses (synaptogenesis), and the creation of new blood vessels in the brain (Buonomano and Merzenich, 1998; Cotman and Berchtold, 2002; Dong and Greenough, 2004; Ming and Song, 2005; Voelcker-Rehage and Willimczik, 2006; Ponti et al., 2008). Brain plasticity is a lifetime developmental process and continues to play a significant role in older adulthood. Cognitive and motor activities have to be intellectually stimulating and physically appropriate to bring about maximal benefits to the aging brain (Colcombe et al., 2003, 2006).

The increasing number of older adults and those who develop MCI or AD pose great challenges to our society (Brookmeyer et al., 2007). Effective interventions must be taken to reduce the impact of functional decline. We summarize recent knowledge of brain plasticity and highlight the applications of developmental studies on cognitive training and movement therapy. We discuss the close relationship between neural plasticity, motor learning and cognitive aging. The goal is to integrate empirical results focusing on motor skills acquisition and the benefits of motor practice or exercise in older adults.

## Neural plasticity

Cognitive and motor development plays an essential role in human brain maturity and a wide variety of daily functions (Elbert et al., 1995; Barnett et al., 2009). To make better sense of the close interactions between cognitive and motor skills across the lifespan, identifying the biological and environmental factors of human development is of particular interest (Quartz and Sejnowski, 1997; Diamond, 2000; Johnson, 2003; Ronnlund et al., 2005). Over the past few decades, researchers have made substantial efforts and progress in understanding the brain mechanisms of functional changes and structural reorganizations in people at varying developmental stages. Lifetime cognitive and motor development is closely related to each other (Samuelson and Smith, 2000; Yan et al., 2000; Johnston et al., 2001; Salthouse and Davis, 2006). Essentially, brain plasticity, neural maturation and cognitive development play an important role in cognitive and motor learning (Ungerleider et al., 2002; Lacourse et al., 2004; Wright and Harding, 2004).

*Neural plasticity* (also known as neuroplasticity, brain plasticity, cortical plasticity, cortical re-mapping) is an inherent *characteristic* or *ability* for lifelong skills learning and relearning. Specifically, neural plasticity refers to the capacity of the central nervous system (*CNS*) to alter its existing cortical structures (anatomy, organization) and functions (physiological mechanisms or processes) in response to experience, learning, training, or injury (Hubel and Wiesel, 1970; Kolb and Whishaw, 1998; Wall et al., 2002; Kolb et al., 2003; Ballantyne et al., 2008). When describing neural structures and functions, the cortical sensory organizations are usually portrayed as mappings where specific sensory inputs project to given cortical locations to form sensory-based neural representations (Buonomano and Merzenich, 1998). When an individual acquires novel skills or information, the newly obtained experience will alter the neural maps, networks, pathways or circuits made up of countless neurons and synapses (Wall et al., 2002). Neural plasticity is, therefore, a biological foundation of the learning brain (Taubert et al., 2010).

Experience-dependent changes in the lower neocortical regions can reshape the activation patterns and the anatomy of the cerebral cortex (Wall et al., 2002). Sensory input, knowledge and motor learning activities stimulate cortical changes (Rakic, 2002; Taubert et al., 2010). In skill learning or repeated exposure to stimulations and experiences, relevant neurons often “fire together [and] wire together”. The associated neurons of a given response will be activated simultaneously in response to similar stimuli in the future. Learning endeavors or experiences modify the existing cortical structures or mechanisms via neurogenesis, gliogenesis, or by changing the strength of inter-neuronal connections (synaptogenesis) (Buonomano and Merzenich, 1998; Cotman and Berchtold, 2002; Dong and Greenough, 2004; Voelcker-Rehage and Willimczik, 2006; Ponti et al., 2008).

Learning-dependent neural plasticity is a lifetime developmental process in which age-related *critical* *periods* or *time windows* seem to exist for significant cortical reorganization (Buonomano and Merzenich, 1998; Cotman and Berchtold, 2002; Rakic, 2002; Dong and Greenough, 2004; Voelcker-Rehage and Willimczik, 2006; Ponti et al., 2008). During the critical periods in early life, among competing sensory inputs of a given experience, neural representation of the most critical aspect is formed and *consolidated* (*e.g*., the hippocampus helps the formation of long-term memories) (Hensch, 2005). In contrast, experiences may induce fewer changes during non-critical periods of brain development. Young children may gain more from skill learning during the age-sensitive periods than their older peers (Ito, 2004). Developing brains may have a greater potential to be trained or are more flexible in skills learning than developed brains (Kolb, 2000; Bunge and Wright, 2007). Training or treating children with certain developmental disorders during sensitive periods can maximize the benefits of neural plasticity and skill learning, which is important for their rehabilitation (Yan et al., 2002; Ito, 2004).

Sensory networks may be immutable beyond the critical periods of cortical plasticity (Ponti et al., 2008). However, experiences still enhance cognitive and motor functions via synaptogenesis and neurogenesis in certain brain areas, such as the hippocampus and cerebellum. Brain plasticity may be an age-related, age-dependent, or age-independent developmental process (Kolb et al., 1998). Instead of being a unitary process, experience-based neural plasticity is dynamic: varying as a function of cortical regions, time, and types of sensory or motor learning (Quartz and Sejnowski, 1997; Johnson, 2003). Understanding experience-related neural plasticity within a developmental framework along with the optimal timing of skills training or rehabilitation for individuals at different ages certainly has clinical and educational relevance. With the understanding of brain plasticity, for instance, clinicians can effectively assess and treat developmental disorders (*e.g.*, developmental coordination disorders (DCD), and developmental dyspraxia, a kind of motor learning difficulty in children) and aging-related dysfunctions (*e.g*., MCI, AD, or dementia). Instructors can teach developmentally appropriate skills to children (Yan et al., 2002; Ito, 2004). Without doubt, there are pressing and increasing demands for the development of more effective approaches for older adults who suffer from cognitive or motor deficiencies (Yan and Zhou, 2009; Belleville et al., 2011; Hill et al., 2011; Ren et al., 2013).

The next question concerns the length of learning that can induce activity-dependent neural plasticity in *macro*- or *micro*-*structures*. Developed brains may restructure themselves due to extensive or rigorous skill learning lasting months or following a short period of practice (Taubert et al., 2010). A remarkable increase of gray matter volume was observed in the prefrontal, frontal and parietal regions while the learners were acquiring a balance-control skill. Changes in fractional anisotropy of the related white matter were observed following skill learning. Even minor adjustments in the training experience result in structural modifications and the rebuilding of functional capacities. Furthermore, a few weeks of training in mice led to the formation and removal of synapses, and a heightened level of synapse turnover. These changes may collectively cause an adaptive remapping of neural pathways (Trachtenberg et al., 2002). When a given stimulus is repeatedly and intentionally coupled with the consistent activation patterns of a set of neurons during the critical period, the neural representation of that stimulus is reinforced (Zhou and Merzenich, 2007).

Therefore, activity-dependent neural plasticity can be induced by both lengthy-extensive and brief-intensive practice (Ziemann et al., 2004). In learning a complex visuomotor task, older and younger learners showed similar increases in corticomotor excitability after several minutes of training, reflecting similar cognitive-motor plasticity of the two age groups (Cirillo et al., 2011). When younger adults actively engaged in a learning and memory task for months, the size of gray matter expanded remarkably in the posterior and lateral parietal sites (Draganski et al., 2006). Task-specific memory capacity, developed via a dynamic process of information encoding and retrieval, results in greater functional plasticity in the multi-level memory networks. For elite athletes, superior sport expertise and performance are the outcomes of long-term training and cortical-spinal plasticity (the “Olympic brains”) (Nielsen and Cohen, 2008). Sport-specific experiences and the resultant neural enhancements contribute to an effective use of sensory information in judgments or decision-making and better motor memory consolidation in expert players.

Mental exercise is also reported to have a positive impact on long-term neural activities (Lutz et al., 2004). For example, adaptive working memory training increased neural efficiency in working memory tasks in older adults (Brehmer et al., 2011). Working memory training induced white matter increases that were correlated with performance improvements in older adults (Engvig et al., 2012). In addition, multitasking was enhanced in older adults after video game training. The benefits can be transferred to non-trained sustained attention and working memory tasks (Anguera et al., 2013). It is thought that complex training environments (*e.g.*, video games) are a key for successful cognitive training and interventions (Bavelier et al., 2012).

Evidence shows that experience-dependent neural plasticity can be stimulated both by physical and mental practice to different extents, depending on the training contents and the abilities to test. Compared to physical training, greater improvements in executive functions were observed after cognitive training (Gajewski and Falkenstein, 2012). Cardiovascular and coordination training led to differential changes in sensorimotor and visual-spatial networks (Voelcker-Rehage et al., 2011). These observations are important for both cognitive improvements and motor learning.

## Motor learning

Motor learning is conceptualized as the process of deliberate or goal-directed practice that results in long-lasting performance stabilization and adaptation (Schmidt and Lee, 2005). Motor learning and relearning are lifelong activities directly associated with experience-related brain plasticity (Ungerleider et al., 2002; Voelcker-Rehage and Willimczik, 2006; Taubert et al., 2010). Recent studies reported that motor skills can be learned both in *on-* and *off-line* modes. On-line learning (also known as *within-session* practice-dependent acquisition) occurs when a learner practices a given skill or a set of skills. Upon completion of the task-specific practice, a learner continues to acquire or stabilize a given skill or a set of skills. In particular, motor learning would be more pronounced after an optimal post-practice interval during which a learner is sleeping or napping. This mode of motor learning refers to off-line learning (also known as *between-session* practice-independent performance enhancement). Nighttime sleeping or daytime napping is required for such a learning mode. In motor behavior literature, motor skill acquisition is said to be composed of these two closely related learning mechanisms (Newell, 1991; Sanes, 2000, 2003; Thomas et al., 2000; Cohen et al., 2005; Yan et al., 2009; Debarnot et al., 2011; Wilhelm et al., 2012). Both practice and sleeping facilitate motor skills learning.

Motor learning takes place when a learner repeatedly and actively engages in physical or mental practice of a given skill or a set of skills (Schmidt and Bjork, 1992). Researchers of on-line learning primarily examine how learners of different ages or skill levels, in different learning conditions, acquire a variety of motor or cognitive skills *during practice*. If on-line learning occurs, skill improvements can be observed during the practice phase and can be captured in immediate or delayed retention or transfer tests. Over the last few decades, substantial evidence has shown that on-line learning benefits children and younger adults (Thomas et al., 1979; Newell, 1991; Deutsch and Newell, 2001, 2005; Schmidt and Lee, 2005; Magill, 2011). Despite having cognitive-motor *deficits* (Yan, 2000; Schaie, 2004; Dennis and Cabeza, 2008), older learners are able to use skill feedback (*e.g.*, knowledge of results *(KR)*, feedback about the outcome of a motor skill) for motor learning to an extent similar to that of younger adults (Swanson and Lee, 1992; Carnahan et al., 1993, 1996; Wishart and Lee, 1997; Schiffman et al., 2002). However, Liu et al. (2013) recently showed that older learners with cognitive-motor deficiency demonstrated limited on-line learning as reflected in small improvements of movement time (*MT*, time difference between onset and offset of an action) and small reductions in timing error (*TE*, time gap between the target time and MT). In retention tests, older adults with intact cognitive and motor functions outperformed those who had relatively poorer scores in an eye-hand-coordination test (finger tapping), the Mini Mental State Exam (*MMSE*), and the Trail Making Test (*TMT-*A and B). Cognitive, neural and motor deficiencies in older adults might explain their compromised on-line motor learning (Albert, 1997; Seidler, 2006; Yan et al., 2008; Yan and Zhou, 2009; Ren et al., 2013).

One important finding of Liu et al. (2013) was that the interval between each learning trial and the delivery of KR, and the interval between the delivery of KR and the next learning trial, were critical for successful on-line motor timing. For cognitively unhealthy older learners, longer intervals (6, 12 s) reduced the beneficial effect of KR on on-line learning to a greater degree than on the learning of their healthy counterparts and younger adults. Poorer attention, concentration and working memory, and decreased information processing speeds in older adults may be the reasons. For instance, cognitive aging is typically associated with reductions of attention and memory capacities (Salthouse, 1996; Chao and Knight, 1997; Maylor, 1998; Maylor and Lavie, 1998; Johnson et al., 2002; Hedden and Gabrieli, 2004; Anguera et al., 2011). Older learners might fail to register the given KR or keep the action-related information *active* for a long period of time. This working memory problem is the “*binding deficit”* of older learners in using KR for on-line learning (Johnson et al., 2002). Furthermore, Anguera et al. (2011) attributed the reduced learning efficiency in older adults to their inability to couple KR for a given trial with the neural representation of the skill. Interactions of cognitive and motor aging, environment and neural processes collectively contribute to the observed differences in motor learning between older and younger adults. In the case of cognitively healthy older adults, without additional practice, their newly acquired motor experiences or memories could be retrievable for a minimum of 2 years after learning (Smith et al., 2005). Thus, aging is not necessarily associated with memory deterioration, but aging-related cognitive-motor dysfunction plays a central role in the decline of on-line motor learning.

Based on the results, a subsequent question to consider is how motor training (on- or off-line) strengthens memory and executive functions that are crucial for older learners suffering from functional decline (Baddeley, 1996, 2002; Anderson et al., 1998; Seidler, 2007). The second question is whether sleep-based off-line motor learning enhances brain functionality of older adults. The third question is how physical exercise, motor practice and skill learning improve motor performance and brain fitness based on the neural plasticity mechanisms aforementioned. Perhaps off-line learning is more profitable than on-line learning in motor learning in older learners. There is less interference with learning during sleep; the off-line learning mode may allow sufficient time or cognitive resources to process and consolidate skill-related information into long-term memories (Yan et al., 2009).

While motor skills become better during practice sessions, skills continue improving between practice sessions, particularly during post-practice sleeping (Walker et al., 2003; Walker and Stickgold, 2004; Walker, 2005, 2009). Different neural mechanisms for over-day and over-night off-line learning modes may exist (Robertson et al., 2004, 2005). Contrary to our expectation that sleep-based learning would be more advantageous than on-line learning, older learners showed small gains in skill retrieval after sleep (Yan et al., 2009). Brown et al. (2009) suggested that over-night sleeping had little benefit for motor learning in older adults with intact motor or cognitive functions. The findings of Yan et al. (2009) were inconsistent with the learning effects observed in children or young adults and might suggest a reduction of neural plasticity in older adults (Thomas et al., 2000; Wilhelm et al., 2008, 2012; Yan et al., 2009). Discrepancies of results are possibly due to quality, timing, or duration of sleep, experimental design or interference from other experiences (Rickard et al., 2008; Cai and Rickard, 2009). For example, during the 24 h post-practice interval, there was no control over the activities or schedules of the learners, which might have affected the learning of older adults more than that of their younger peers (Brown et al., 2009). Greater individual differences in the cognitive and motor capacities of older adults may also hinder their on- and off-line motor learning (Yan et al., 2009). Subsequent research is warranted to clarify these issues.

Finally, we discuss how and why physical practice and mental training improve motor skills and brain fitness based on the concept of brain plasticity. Two essential questions need to be answered: what are the neural changes associated with motor learning? How does neural plasticity contribute to motor learning? From a biological viewpoint, skills learning or relearning is associated with neural plasticity for the survival and development of a species. Structural and functional alternations take place in the neural system throughout the lifetime (Ungerleider et al., 2002; Voelcker-Rehage and Willimczik, 2006; Taubert et al., 2010). Motor learning results in neurobehavioral changes, making the neural system shift from a conscious control (central, command-driven, top-down) to an unconscious (peripheral, feedback-based, button-up) control mode; the neural representations of the skill are formed during this process (Willingham, 1998). The flexible or changeable nature of cortical reorganization is the basis of skill learning.

Physical exercise brings about changes to brain structures and functions. An animal study suggested that physical exercise or motor learning can increase the thickness of the motor cortex (Anderson et al., 2002). An increased density of blood vessels and the development of synapses in the cerebellum were observed following physical exercise (Black et al., 1990). Physical exercise contributes to memory formation and increases the number of neurons and synapses (dentate gyrus) in both young and old mice (van Praag et al., 1999a,b, 2005). Deliberate training, or changing the surrounding environment, enhances cortical reorganization and functions that facilitate memory formation in rats through altering gene expression and the concentration of the brain-derived neurotrophic factor, BDNF (Briones et al., 2004; Farmer et al., 2004; Berchtold et al., 2005). The benefits of physical and mental training are largely mediated by enhanced BDNF signaling (Korol et al., 2013); whereas increased temporal lobe connectivity after aerobic training is associated with BDNF, insulin-like growth factor type 1 (IGF-1), and vascular endothelial growth factor (VEGF; Voss et al., 2013).

Environmental factors such as exercise, training, injuries and rehabilitation change neural plasticity by varying degrees in different cortical regions. As a result of long-term professional training, the cerebral structures and functions of expert musicians show significant changes. The cortical alterations may start in the early stages of training, or life, and develop continuously in higher regions later, reflecting rapid and remarkable changes in the critical period of neural plasticity (Schlaug, 2001). In humans, intentional exercise and cortical plasticity are closely related; exercise contributes to better memory by elevating BDNF (Vaynman et al., 2004, 2006). At the behavioral level, older adults demonstrated “*motor plasticity”* or the ability to acquire new motor skills to an extent similar to that of younger peers (Roller et al., 2002; Voelcker-Rehage and Willimczik, 2006). Skill differences between younger and older adults in motor learning should reflect the ability of CNS to reduce neural noise and coordinate agonist-antagonist muscle activities, but not older adults’ inferior potential to learn or relearn (Christou et al., 2007).

Furthermore, computer-based practice significantly enhances cognitive rehabilitation in healthy older adults; the beneficial effects of training can be sustained for 3 months. The results bear important implications about the “*reversibility”* of older brains (Mahncke et al., 2006). Injury studies support the notion of structural and functional reversibility of the higher regions of CNS. For instance, certain therapeutic treatments or training can repair or recover the vanished, or reduced, brain functions caused by injury (Stein and Hoffman, 2003; Wieloch and Nikolich, 2006). Given the potential value of plasticity-based skill learning and the contribution of motor practice to functional neural regeneration, these studies have important implications for older adults. Specifically, older adults normally suffer from functional deficiencies. The plasticity-based motor learning or training should integrate multiple tasks or activities to be implemented at multiple skill levels (fundamental, daily, motor, and special skills) and with multisensory inputs (Nojima et al., 2012). Training regimens should be specific, function-oriented, precise, simple, and combined with daily tasks (*e.g*., locomotion, balance control or fall prevention). Individual differences in cognitive and motor functions in older adults should be considered when designing and implementing training programs. Future research to identify major barriers and facilitators to brain fitness and motor functionality in older adults is integral to cognitive-motor skill learning and rehabilitation. An apparent challenge now lies in the search for an optimal therapeutic approach to enhance skills learning in older adults.

## Cognitive aging

While discussing brain fitness or motor ability of older adults, we describe the developmental pattern and define “normal aging”, and “cognitive aging”. We will also answer the questions, “what is successful aging?” and, “how do older adults achieve successful aging through exercise or practice?” Importantly, we address the features of learning and relearning in older adults who are cognitively and motorically intact or impaired. Finally, the links between physical exercise and brain function in older adults will be highlighted. 

**Figure 1:**
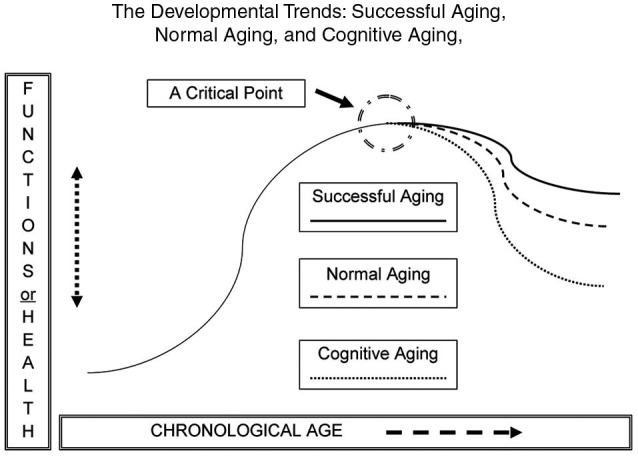
**The likely developmental trends across the human lifespan (an inverted U-shape)**. The down-turning paths are for normal aging, cognitive aging, and successful aging in motor and cognitive functions. Overall, the functional curves are moving downwards during older adulthood. The slope of functional change is subject to a number of biological and environmental factors.

Figure 1 shows an inverted U-shaped pattern of motor and cognitive ability and three possible descending paths in older adulthood: normal aging; cognitive aging; and successful aging (Yan et al., 1998, 2000; Yan and Zhou, 2009). Functional capacity increases during childhood and young adulthood and decreases in older adulthood. The slope of functional changes largely depends on individual differences in genetics, personality, motivation, lifestyle, socio-cultural background, exercise opportunities and learning experiences. Variations in the curves are collective or interactive outcomes of these biological and environmental factors.

From a lifespan developmental viewpoint, *normal aging* or *aging* literally means that an individual may experience multiple aspects of expected change (*e.g.*, neural, biological, physiological, psychological or social) during the passage of time, from conception to death. A functional increment or progression can be seen in early life (childhood, youth, and young adulthood), and even in older stages; regression takes place at later stages (Bowen and Atwood, 2004). *Cognitive aging* is a set of gradual and functional regressions of cognitive abilities (*e.g*., attention, memory, reasoning, decision-making and processing speed; Salthouse, 2004). Steady decline of cognitive ability can be observed across the lifespan, and it accelerates after young or middle adulthood (Salthouse, 2009). Older adults also experience a major decline in movement functionality, such as reduced walking speed, poorer eye-hand coordination, and compromised learning abilities (Yan, 1999, 2000; Yan et al., 2009; Studenski et al., 2011).

Functional deteriorations in cognitive and motor domains are of the greatest concern to many older adults and the people around them. However, Figure 1 also shows the third and optimal path of development in the “getting older” process. This ideal course of development is *successful aging*, owing to the contributions of neural or cognitive *reserves* of older adults with an active lifestyle (Arbuckle et al., 1998; Stern et al., 2003; Daffner, 2010). Contrary to the greater functional decline in older adults in the categories of “cognitive aging” or “normal aging”, those in the category of “successful aging” suffer less from declining abilities and enjoy a relatively higher level of *cognitive wellness* through the reorganization of brain networks (Sala-Llonch et al., 2012). The main characteristics of successful aging are “low probability of disease or disability, high cognitive and physical function capacity, and active engagement with life” (p. 433), along with others such as having greater life or emotional satisfaction, enjoying more learning opportunities, and participating in active social and intellectual activities (Rowe and Kahn, 1997; Chou and Chi, 2002; Strawbridge et al., 2002; Menec, 2003; Duay and Bryan, 2006).

Motor and cognitive functions seem to be fairly *homogeneous* in early life and gradually become more *heterogeneous* as humans approach the hypothetical “critical point”, and thereafter (Figure 1). Variations of cognitive and motor capacity lead to three possible trends in older adults (Rowe and Kahn, 1987; Yan et al., 2000). Attaining the state of successful aging is the goal to maximize the quality of life and reduce the negative impact of cognitive aging in older adults (Rowe and Kahn, 1987). Here we focus on the benefits of potential applications of training-induced neural plasticity in successful aging.

**Figure 2:**
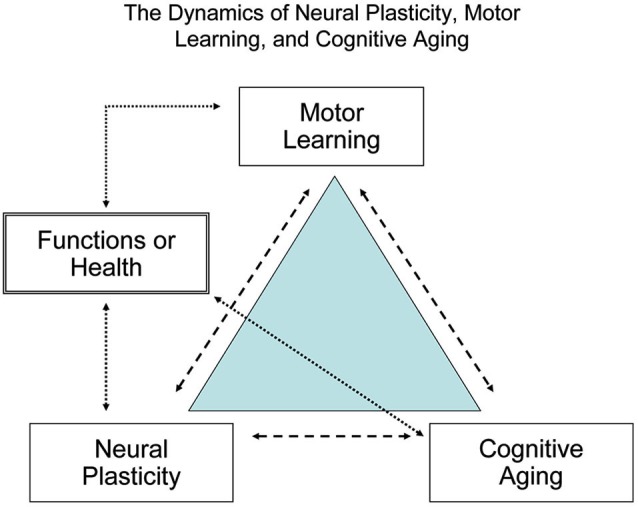
**The dynamic relationship between brain plasticity, motor learning, and cognitive aging.** To maximize human brain fitness and motor functions signaled by the quality of life and independence in daily activity, habitual cognitive and motor learning or practice is required across the lifespan, particularly for older adults.

Figure 2 shows the dynamics of neural plasticity (the biological foundation for learning), motor learning or relearning (the process of acquiring or consolidating new or learned skills), and cognitive aging (the deteriorating capacities). Interactions of cognitive aging, experiences, environments and brain development collectively contribute to the observed discrepancies in motor and cognitive functions between younger and older learners. To achieve successful aging, regular cognitive and motor training is essential throughout the lifespan and especially for older adults who are at high risk of functional deterioration (Kramer et al., 2000, 2006).

Over the past three decades, there has been a large volume of research to delineate how exercise or motor practice improves neurocognitive function or wellness in older adults (Colcombe and Kramer, 2003). Of particular interest are the studies concerning how the brain responds to physical exercise or skill learning to improve neurocognitive functions in older adults.**** Kramer et al. (2006) reviewed cross-sectional, longitudinal and interventional research into the relationship between physical fitness and brain function in older adults (Kramer et al., 2006). The beneficial effects of aerobic fitness on executive functions and working memory in older adults were supported by several subsequent studies (Carlson et al., 1999; Yaffe et al., 2001; Barnes et al., 2003, 2007; Studenski et al., 2006). The benefits of vigorous exercise and motor learning for improving neurocognitive functions in older adults can be explained by one or all of the three theoretical accounts: information processing, executive functioning, and memory development (Clarkson-Smith and Hartley, 1989; Tomporowski and Hatfield, 2005). The robust relation between exercise and cognition apparently requires additional clarification: (1) exercise participation may contribute more to executive processes than to the other two domains (information processing and memory development); (2) genetic factors may play a critical role in identifying the target population at risk of cognitive impairment; and (3) the measurement of differences between subjects is critical. Brain processes are not often observable or detectible at behavioral level. However, neuroimaging techniques can show small changes in neural metabolism or activation to indicate major differences as the result of exercise or skills learning for neurocognitive functions.

Although studies have suggested the positive effects of exercise on cognitive functions in older adults, one of the prevailing problems was that many studies were cross-sectional ignoring time to time performance changes. A need for longitudinal-interventional studies became apparent. For instance, Yaffe et al. (2001) reported that when community-dwelling older women took cognitive tests 6 years after baseline tests, those who had higher physical activity levels showed less cognitive decline (2001). A decade-long study also showed that poorer fluid intelligence was associated with lower levels of physical activity; those who reported the lowest levels of activity in middle age were at the highest risk of cognitive decline later in life (Singh-Manoux et al., 2005). In a study over the course of 12 weeks, older adults in the walking group outperformed, in inhibitory control and selective attention, those who were not physically active. Therefore, a sustained exercise program can protect against cognitive decline in older adults (Kamijo et al., 2007).

Another question to ask is, “how can the required training or exercise (exercise modality, intensity, duration and frequency) be implemented in a meaningful and effective way to help older adults?” Some researchers classified research on exercise and brain health into four categories: (1) to determine frequency, intensity, type and level of physical activity for diverse aging populations; (2) to identify the major or realistic cognitive benefits by participating in physical activity; (3) to understand the psychological and physiological factors that influence physical activity participation both in healthy and unhealthy older adults; and (4) to promote appropriate interventions, establish practical evaluation criteria and develop policies to increase physical activity among those at risk of cognitive impairment (Prohaska and Peters, 2007). With the outcome of these studies, researchers can bridge the gap between research and the implementation of physical activity for *brain health*. Of course, the difficult question of how to implement exercise still remains to be answered; however, these studies offer some of the most important guidelines for physical exercise and behavioral enhancement in older adults.

Brain functionality has long been attributed to the maintenance of independence and a high quality of life in older adults (Kramer et al., 2000). The preservation of neurocognitive function during aging is crucial for maintaining the required capacities of neural pathways and cortical networks that control cognition and motor behaviors (Yan and Dick, 2006; Yan et al., 2008). Brain health or fitness is crucial for cognitive and motor functioning in older adults, and for protection against pathogenesis of neurodegenerative diseases such as AD (Cotman and Berchtold, 2002). For example, short-term intensive exercise or long-term regular training improves learning while preventing the progression of AD in mice by increasing BDNF protein and decreasing extracellular amyloid-β plaques (Adlard et al., 2004, 2005). In large-sample longitudinal investigations, Laurin et al. (2001) and Barnes et al. (2003) suggested that physical exercise could protect older adults from cognitive deterioration and, possibly, from dementia. Exercise improves vascular function, decreases obesity and reduces inflammatory markers to enhance brain health and functioning (Laurin et al., 2001; Barnes et al., 2007). Equally important, exercise prevents loss of neurons or neural dysfunction that ultimately leads to the development of dementia (Verghese et al., 2003, 2006; Barnes et al., 2007). Physical activity also helps reduce cognitive decline associated with long-term hormone replacement therapy in menopausal women (Erickson et al., 2007). Both exercise and environmental enrichment can ameliorate hippocampal aging by restoring neurogenesis and neuroimmune cytokine signaling in aged mice (Kumar et al., 2012; Speisman et al., 2013a,b).

Regarding the effects of exercise on *brain plasticity*, Colcombe et al. (2004) tested older adults ranging from 58 to 77 years in a cross-sectional study with VO_2_ (oxygen consumption), reaction time, cognitive tests, functional MRI (*f*MRI) measures. Very fit older adults outperformed those less fit in behavioral and neuroimaging parameters. Better cardiovascular fitness resulted in superior test performances on executive functions, suggesting exercise-dependent neural plasticity of the aging brain. By improving physical fitness, neural structure, synaptic plasticity and transmission can be strengthened.

Finally, we discuss our two experiments exploring motor control and learning in MCI and AD patients. MCI usually refers to the transitional or intermediate phase between cognitive aging and AD. Older adults with MCI are increasingly at risk of developing dementia (Petersen et al., 1999, 2000; Petersen, 2000). In the first study, we examined whether deliberate training can improve functional control and motor performance in healthy older adults and their AD and MCI peers (Yan and Dick, 2006). A ballistic aiming arm movement was the task to learn and test. Differences in MT, jerk (movement smoothness), and the percentage of primary sub-movement (a reflection of top-down or motor planning control) between subject groups across six blocks of tests provided critical evidence concerning motor control mechanisms. Surprisingly, as a result of motor learning, AD and MCI subjects demonstrated an increased programming control and more extended improvements in MT and movement smoothness than their healthy counterparts. In the subsequent study, a handwriting task was used to explore whether AD or MCI had impaired fine motor actions primarily controlled by small muscles or muscle groups (Yan et al., 2008). Results showed that cognitive disorders reduced the control efficiency of fine motor tasks in complex settings (combining wrist and finger movements), but not in simple settings (only requiring wrist or finger movements). The findings of both studies may not be directly associated with the effects of motor learning on brain health or neural plasticity. However, we certainly know more about how cognitive disorders impair motor control, its possible underlying mechanisms, and potential therapeutic interventions for affected older adults.

## Future research

The human lifespan continues to increase and the general population is aging quickly. We highlight key theories and related research into neural plasticity and the application to cognitive training and movement therapy in older adults. We discuss the close relations between the neurodevelopmental characteristics of brain plasticity, motor learning and the functional changes associated with the aging processes (cognitive and successful aging). We emphasize the benefits of motor practice or physical exercise for both cognitively normal and impaired older adults. We propose ideas to enhance future geriatric research or practice for older adults. We suggest using off-line sleep-based motor learning and mindfulness training for older adults who may suffer from cognitive or motor disorders.

More attention should be paid to the contribution of sleep-based skills learning to cognitive and motor skills, and possibly to the physical and neural rehabilitation of older adults (Walker et al., 2003; Robertson et al., 2004, 2005; Walker and Stickgold, 2004; Walker, 2005, 2009; Yan et al., 2009). Although research in this area seems inconclusive, in that reduced capacity of off-line learning is sometimes observed in older learners, we believe that older adults should take advantage of their sleep (or nap) as a supplemental means to develop greater brain fitness. In fact, a number of factors may have confounded the reported insignificance of sleep benefits in older adults (Brown et al., 2009; Siengsukon and Boyd, 2009; Yan et al., 2009). Time-of-day of sleep (the circadian effect) or homeostasis (time since last sleep) may reduce memory consolidation and play an unknown part in off-line motor learning in older adults (Rickard et al., 2008; Cai and Rickard, 2009). Individual differences in cognitive and motor functionality in older adults may decrease the gain from sleep-based learning (Yan et al., 2009). Variation of sleep habits in older adults is an important factor requiring control in future research. Off-line learning or memory consolidation may contribute to older adults’ cognitive and motor functionality. For instance, cuing or re-arousing existing experiences during sleep may be used to facilitate memory consolidation (Rasch et al., 2007; Rudoy et al., 2009). These approaches may be worth trying in older adults.

Furthermore, mindfulness training or awareness meditation may also benefit cognitive and motor rehabilitation in older adults. Mindfulness training refers to the mind and body exercises that facilitate attention focus on the experience of current time. It helps participants reach internal peacefulness, mind-body integration or coupling, via meditation (Siegel, 2009). Importantly, the inner experience of mindfulness improves brain function, reminiscence and social connections, thereby contributing to better mental and physical wellness (Hölzel et al., 2008, 2011). In this regard, mindfulness training has clinical implications for cognitive or motor therapy in older adults. However, the mechanisms underlying the benefits of mindfulness training are still open to discussion. Mindfulness exercise may be useful to increase neuro-protection that older adults need.

Finally, we have three important points. The first is that participation in exercise is critical for preventing cognitive decline in older adults. Persistence maximizes the gains from physical activity (Neely and Bäckman, 1993; Dik et al., 2003). Secondly, physical activity should be realistic and easy to accomplish for older adults (*e.g*., deliberate walking, mind games, or leisure activities; Willis and Nesselroade, 1990; Hultsch et al., 1999; Scarmeas et al., 2001; Schooler and Mulatu, 2001; Ball et al., 2002; Wang et al., 2002; Wilson et al., 2002; Weuve et al., 2004). Last but not the least, as the use of technology becomes increasingly prevalent, more computer-driven technologies will be utilized in training or rehabilitating older adults. In recent years, computerized training has demonstrated promising benefits for improving memory, which can be maintained beyond the training period (Zelinski et al., 2011). The development of such training programs would definitely contribute to better cognitive and motor skills for older adults in future. All activities for older adults may enhance their cognitive-motor functionality due to brain plasticity and, eventually, increase their quality of life over a long period of time.

## Conflict of interest statement

The authors declare that the research was conducted in the absence of any commercial or financial relationships that could be construed as a potential conflict of interest.
